# Research attitudes in families of individuals with Down syndrome: importance for clinical trials

**DOI:** 10.1186/s13195-022-01120-6

**Published:** 2022-11-23

**Authors:** Ira T. Lott, Katharine A. Kirby, Eric Doran, Joshua D. Grill

**Affiliations:** 1 Department of Pediatrics, University of California, Irvine, Orange, CA 92868 USA; 2 Center for Statistical Consulting, University of California, Irvine, CA 92697 USA; 3 Institute for Memory Impairments and Neurological Disorders, Department of Psychiatry and Human Behavior, Department of Neurobiology and Behavior, University of California, Irvine, USA

**Keywords:** Down syndrome, Alzheimer disease, Trisomy 21, Research attitudes, Clinical trials, Survey, DS-Connect

## Abstract

**Background:**

Individuals with Down syndrome (DS) are increasingly eligible for clinical trial intervention, particularly for the treatment or prevention of Alzheimer disease (AD). Yet, little is known about research attitudes that may contribute to decisions regarding clinical trial enrollment for people with DS, a gap which is addressed in the current study.

**Methods:**

The *Research Attitudes Questionnaire* (RAQ) is a brief validated instrument that measures cultural and social factors which influence clinical trial enrollment decisions in the general population. Applied herein to a cohort of 1002 families who have an individual with DS, this survey was carried out through a national registry (*DS-Connect*). In addition to the RAQ, demographic data were collected.

**Results:**

The response rate to the survey was 49.9%. Respondents were asked to complete demographic information and to respond to the 7 question RAQ. The scores were stratified by a cut point assigned a priori into those more favorable toward research participation vs. those less favorably inclined. Within this sample, nearly 95% self-identified as the primary caretaker for the individual with DS. The RAQ score analyses generally indicated favorable respondent views toward research with particularly high favorability ratings from respondents who had previously participated in research and from those who were older (*P* = .01 to .001).

**Conclusions:**

This is one of the first formal studies to evaluate research attitudes among relatives of individuals with DS and shows the feasibility of using this approach to answer important questions that will guide trialists developing treatments for AD in DS*.* Future research will require broadening the racial and ethnic mix of respondents and the role that a standardized assessment of research attitudes will have for clinical trial participation.

## Background

Individuals with Down syndrome (DS) are at high risk for Alzheimer disease (AD) and are increasingly eligible for clinical trials to slow, ameliorate, and even prevent AD symptoms [1–3]. When faced with a clinical trial opportunity, adults with DS may struggle to understand the risks, benefits, and complexity of study protocols, thus potentially rendering them unable to provide legally informed consent. The elements of consent require adequate provision of information, freedom from coercion and undue influence, and decisional capacity. Even in the setting of AD prevention trials, people with DS who are cognitively asymptomatic may look to family members and other legally authorized representatives to assist in trial enrollment decisions. Despite the important and growing attempts to include people with DS in AD research, little is known about attitudes that may influence trial participation. Failure to understand attitudes towards research by legally authorized representatives of people with DS may contribute to the larger issue of under-representation of people with intellectual disability in randomized clinical trials [4].

Attitudes toward research, and toward clinical trials specifically, have been extensively studied in the general (non-DS) population at risk for AD. Several such studies have incorporated the *Research Attitudes Questionnaire* (RAQ), a brief validated instrument developed to measure social and cultural factors that influence research enrollment decisions [5]. The RAQ has shown good internal validity and reliability in the general population at risk for AD [5] and is associated with willingness to participate in clinical trials [6–8]. However, these considerations are unknown for adults with DS and their families.

Advances in the development of treatment approaches that would improve patient independence, cognition, and the amelioration of neuropsychiatric symptoms are a major focus for clinical trials for AD in the general population [9]. However, there remains a need for dramatic improvements in participant recruitment [10, 11]. Additional barriers to clinical trial participation may exist for individuals with intellectual disability (ID), including those with DS [12]. These barriers include numerous tiers of structural organization within the ID community, persistent stigma toward research among some families, accessibility, and caregiving demands [13]. Suggestions to improve recruitment from the population with ID, including those with DS, have comprised more personalized interaction with care providers [14] and the creation of research registries for potential participants [15].

Each of these approaches can be informed by improved understanding of the research attitudes of family members who may be involved in enrollment decisions for people with DS. We undertook a survey study of a large population of such individuals, producing a data set that is among the first to explore attitudes toward research for people with DS.

## Methods

### Participants

This survey was carried out through *DS-Connect*, a registry developed and managed by the *Eunice Kennedy Shriver Institute for Child Health and Human Development*. *DS-Connect* is a resource designed to connect people with DS and their families to researchers and healthcare providers. It is a web-based, voluntary registry that may serve as a resource for research studies [16]. Through this registry, 2100 registrants had agreed at the time of their enrollment to be contacted about potential research opportunities. *DS-Connect* staff sent three email notifications to these potential respondents. There were 1048 responses (a response rate of 49.9%). There were 46 responses that had to be excluded because of incomplete information, leaving a final responding sample size of 1002. The survey was also sent to individuals with DS, but the responses indicated that the information sought by the RAQ proved too complex and unreliable for survey validation. Hence, they (*n* = 22) are not included in this survey report.

### Data collection and management

Respondents were asked to provide check list answers to a series of demographic variables and then to complete the RAQ. The RAQ comprises seven items that measure attitudes towards biomedical research. Specific items include “I have a positive view about medical research in general” (*positive view*), “Medical researchers can be trusted to protect the interests of people who take part in their research studies” (*be trusted*), “We all have some responsibility to help others by volunteering for medical research” (*help others*), “Society needs to devote more resources to medical research” (*societal resources*), “Participating in medical research is generally safe” (*generally safe*), “If I volunteer for medical research, I know my personal information will be kept private and confidential” (*confidential*), and “Medical research will find cures for many major diseases during my lifetime” (*cures lifetime*). Responses to each item of the RAQ were recorded on a 5-point scale from 1 “strongly disagree” to 5 “strongly agree.” RAQ total scores were calculated by summing the scores of the component questions (range: 7–35). We also examined scores for each item.

We assigned an a priori cut point for favorable research willingness. Participants were grouped into those having more favorable (RAQ total scores ≥ 28) or less favorable (RAQ total scores < 28) research attitudes.

The Institutional Review Board at the University of California, Irvine, has reviewed this research activity and have declared it meets the criteria for exempt status.

### Statistical methods

We used descriptive statistics to characterize the sample. Linear regression was used to examine potential associations with total RAQ score. The model included respondent age, education, race, ethnicity, and relationship to the person with DS. We used chi-square tests to identify associations between categorical variables and *T*-tests to compare means of continuous variables. We performed a one-way ANOVA to determine whether there were any statistically significant differences between the means of several independent groups. Linear regression was used to estimate the relationship between total RAQ score and participant characteristics. *P*-values less than 0.05 were considered statistically significant. No adjustments for multiple comparisons were performed. All statistical analyses were performed using the Stata software [17].

## Results

Table 1 lists the demographics of the family members who responded to the survey. Data were not available for individuals invited to participate who failed to respond. Most respondents were female, and 61.4% were over age 50 years. Nearly 95% self-identified as being White race and 96% were of non-Hispanic ethnicity. Nearly 95% of respondents were parents of the person with DS, and 88.9% of respondents identified as the primary caretaker of the person with DS. The average education level of the respondents was high, showing some college or more advanced study in 95.7% of respondents.

**Table 1:** Demographic summary of study participants

Demographic	Freq (%) or mean (SD)
Sex	
Female	880 (87.8)
Male	122 (12.2)
Age, mean (SD)	53.3 (12.1)
Age categories	
< 30	19 (1.9)
30–39	122 (12.2)
40–49	246 (24.6)
50–59	303 (30.3)
60–69	219 (21.9)
70+	92 (9.2)
Race	
White	875 (94.9)
Other	18 (2.0)
Black or African-American	14 (1.5)
Asian	13 (1.4)
American Indian or Alaskan Native	2 (0.2)
Ethnicity	
Hispanic	42 (4.3)
Non-Hispanic	927 (95.7)
Relationship to person with Down syndrome	
Parent	941 (94.5)
Sibling (including full, half or step)	45 (4.5)
Guardian or conservator	4 (0.4)
Other	6 (0.6)
Primary caregiver	
No	111 (11.1)
Yes	887 (88.9)
Education	
Years, mean (SD)	17.8 (3.3)
High school	42 (4.3)
Some college or more	945 (95.7)

The mean (SD) RAQ score among participants was 29.2 (3.8). In a linear regression model for the outcome of total RAQ score, only age was significantly associated with RAQ (estimate = 0.03; 95% CI: 0.01, 0.05; Table 2). None of education, relationship to the person with DS, sex, race, or ethnicity demonstrated a significant association with RAQ in this sample, whether using RAQ as a continuous (Table 2) or bivariate outcome (data not shown).

**Table 2:** Linear regression results for total RAQ score

	Coefficient	95% CI	*P*-value
Age in years	0.03	0.01, 0.05	0.004
Education			
0–12 years	0 (base)	--	
13–17 years	− 0.08	− 1.27, 1.11	0.90
18–22 years	0.28	− 0.91, 1.48	0.64
23–28 years	1.10	− 0.28, 2.48	0.12
Non-Hispanic Caucasian			
No	0 (base)		
Yes	0.83	− 0.05, 1.72	0.065
Relationship			
Parent	0 (base)	--	
Sibling	− 1.04	− 2.17, 0.08	0.07
Guardian/conservator	− 0.22	− 3.92, 3.48	0.91
Other relative	1.45	− 1.58, 4.47	0.35
Sex			
Male	0 (base)	--	
Female	0.07	− 0.64, 0.79	0.84
Primary caregiver			
No	0 (base)	--	
Yes	0.10	− 0.64, 0.85	0.78

In Table 3, the component scores for the RAQ are presented for the overall sample. Scores were generally high; most participants agreed or strongly agreed with each item. The frequency of strong agreement was highest for items assessing participants’ general positive view of research and their belief that society needs to devote more resources to research.

**Table 3:** Frequencies of responses for RAQ items

Score	Strongly disagree	Disagree	Neutral	Agree	Strongly agree
RAQ question					
Positive view	9 (0.88)	6 (0.59)	76 (7.44)	419 (41.00)	512 (50.10)
Be trusted	7 (0.68)	17 (1.66)	132 (12.92)	558 (54.60)	308 (30.14)
Help others	7 (0.68)	25 (2.45)	176 (17.22)	451 (44.13)	363 (35.52)
Societal resources	5 (0.49)	5 (0.49)	82 (8.02)	423 (41.39)	507 (49.61)
Generally safe	5 (0.49)	12 (1.17)	198 (19.37)	556 (54.40)	251 (24.56)
Confidential	5 (0.49)	14 (1.37)	151 (14.77)	487 (47.65)	365 (35.71)
Cures lifetime	9 (0.88)	44 (4.31)	237 (23.19)	403 (39.43)	329 (32.19)

We explored potential differences in research attitudes in groups defined by demographic characteristics. For example, respondents with previous research participation experiences (*n* = 384) had higher RAQ score than those who lacked such experiences (*n* = 613) (mean ± SD, 29.9 ± 3.74 vs. 28.7 ± 3.73; *t*-test, − 4.74, *p* < 0.001).

Exploratory subgroup analyses also supported the observations from the multivariable model. The mean age of respondents answering that they held less favorable attitudes toward research (RAQ < 28) was 52.79, and the mean age of those holding more favorable attitudes (RAQ ≥ 28) was 53.52, a difference of − 0.73 years (95% confidence interval − 2.36 to 0.89; *t*-test, − 0.89; *p* = 0.37). Using age 60 as a cut point, we found that the mean RAQ total score was greater in older (*n* = 311, 29.6 ± 3.88) compared to younger (*n* = 690, 29.0 ± 3.70) respondents (*t* = − 2.5654, *p* < 0.01).

We found no evidence of differential RAQ scores by relationship to the person with DS. Parents (29.2 ± 3.7), siblings (28.2 ± 4.1), and guardians/conservators (29.0 ± 1.8), on average, had similar (*F*(3,992) = 1.42 (*p* = 0.24) and relatively positive attitudes toward research. Similarly, categorizing respondents as having more (RAQ ≥ 28) or less (RAQ < 28) favorable attitudes, we observed no difference in the frequency of categories of respondents. Sixty-nine percent of parents, compared to 64% of siblings and 75% of guardians/conservators had RAQ scores in the higher range (*p* = 0.772).

Non-Hispanic Whites did not differ from other races/ethnicities in their attitudes toward research when responses were grouped together as more vs. less favorable (*p* = 0.098) or when examining RAQ as a continuous variable. Non-Hispanic White respondents had mean RAQ scores 0.8 points higher than participants of other races/ethnicities (29.26 vs 28.42), *p* = 0.065.

## Conclusions

This study provides data indicating the feasibility of administering questionnaires and other studies to elucidate research attitudes, opinions, and preferences among families who have an individual with DS. To our knowledge, the results constitute the first formal study of research attitudes in this population and may be a step towards recognizing the challenges that characterize the conduct of clinical trials in individuals with ID [18, 19]. RAQ has been demonstrated in several studies to be associated with willingness to participate. We viewed the opportunity to collect RAQ in a large sample of DS family members as an important low burden first step to elucidating attitudes toward AD clinical trials in this population.

We found that most of the family members who responded had positive attitudes toward research. Although one of the aims of *DS-Connect* is to address families who have potential interests in clinical trials, enrolling in a registry is not equivalent to participating in a trial. Our sampling method did not allow us to distinguish respondents who would from those who would not support actual trial participation among their family members. Nevertheless, older respondents and those with previous research experiences scored higher on the RAQ, compared to younger respondents and those without previous experiences. These results may indicate that family members who could be involved in decision making about research participation will be supportive of such research endeavors. The results may also indicate that the RAQ has potential as a valuable tool to identify dyads of individuals with DS and their supporters, for recruitment into clinical trials.

The RAQ scores observed here were similar to those observed in samples of individuals who have enrolled in (non-DS) AD research. Previously, the RAQ has predicted willingness to participate in AD research [6] and has distinguished differential willingness between spousal and non-spousal caregivers to persons with AD [20]. Similarly, utility of the RAQ has been demonstrated for predicting behaviors within research studies, namely clinical trial dropout and missing data [21]. Others have found differences in RAQ among racial and ethnic groups [22], seemingly contributing to underrepresentation among some communities in AD research [8]. We did not observe similar differences here, though the number of racial and ethnic minority participants was quite low. Future studies will need to expand ethnic participation in the determination of research attitudes. This need is particularly compelling because of the disparities that exist across the ethnic spectrum in DS regarding the age and cause of death [23, 24]. Other differences between the current sample and previous non-DS research include a lack of association between RAQ scores and respondent education. Sample bias for high education is consistent in non-DS research, and this lack of association may suggest that DS trials could be more inclusive in this regard.

We did observe an association between older age of the family respondents and more favorable research attitudes. This association equated to an approximately 0.15-point difference for every 5 years of age. This observation was somewhat surprising; we postulated that older family members might be more aware and/or negatively affected in their research attitudes by the Willowbrook scandal, which was first exposed in 1971 [25]. In fact, the highest endorsed RAQ item among this sample was a positive view of research in general (Table 3). This may suggest that factors protecting participation of individuals with intellectual disability in clinical research have given family members a broader sense of trust.

While age was associated with RAQ scores, we did not observe an association between RAQ and the relationship to the person with DS, though here too the numbers of non-parent family members completing the survey were likely too limited to detect differences. Further exploration of this issue could be important to future AD trial recruitment since, as parents age, many siblings are increasingly involved in a caregiving role towards their brother or sister with DS [26].

These observations should be considered within the context of previous studies in families who have a member with DS. Among the most important barriers to clinical trials to be identified in individuals with DS is lack of exposure to the potential benefits of research [12]. This finding is supported by our observation that those respondents with previous research experience were observed to have higher RAQ scores. Two possibilities exist to explain this observation: those with the highest RAQ scores participated in part due to their positive attitudes or the experience of participating improved research attitudes in this subset of respondents. In either case, enhancing research education or offering low risk, low burden research experiences (such as this survey) may provide steps to improve research attitudes and facilitate subsequent trial recruitment.

This study also adds to literature exploring consent issues in people with developmental disabilities and some psychiatric disorders [27]. In exploring voluntary consent for research, Roberts [28] highlighted the need to act in accordance with the individual’s sense of what is good, right, and in the person’s best interest in light of their personal history. Structured decision aids seem likely to aid in this process across a wide range of clinical research [29]. In pediatric research, the opportunity to learn more about a condition is a main motivator for parent consent [30]. This may also hold for consenting the more complex medical issues associated with aging in DS and our results may support an openness among family members to playing a role in providing such consent.

In other studies, parents of individuals with DS who were asked if they would approve future molecular approaches to “cure” DS gave complex responses, affected by societal, ethical, age, and caregiving challenges [31]. The results were not always favorable towards the idea of a cure. Yet, opinion was more united for a clinical trial that would improve independence for their loved ones. Parental attitudes towards cognitive-enhancing therapies in DS are determined by safety concerns, logistical considerations, and personal beliefs [32]. The safety element of the RAQ may provide important context for measuring attitudes. Another element of the RAQ reflects the trust that potential participants have in researchers. Perhaps of interest, or opportunity for future interventions to improve attitudes among DS family members, these two items had the lowest frequency of strong endorsement in this sample, though the mean scores were not significantly lower than those observed for the remaining items. It is to be remembered that even if individuals with DS cannot legally consent to research participation, their assent is required prior to each procedure.

Numerous limitations of this study should be noted. While, to our knowledge, this is the first application of the RAQ in family members of individuals with DS, the study is limited in its generalizability by the nature of the sample. Respondents had agreed to join *DS-Connect* and had further agreed to be contacted regarding research endeavors. The great majority of the respondents listed themselves as being of White race and parents of a person with DS. A health disparity exists regarding general knowledge about developmental disabilities in minority race and ethnicity populations, particularly in Latino families who lack English proficiency [33]. Future studies should examine whether research attitudes differ among minority race and ethnicity individuals, compared to non-Hispanic Whites, and sibling and other non-parent family members of people with DS, compared to parents. Finally, our survey included no formal questions about willingness to support a loved one with DS participating in clinical trials (for AD or any other condition). Understanding relationships between RAQ scores and actual enrollment decisions will require further study.

In conclusion, we have completed a novel exploration among a large and unique sample of family members of people with DS. We found that attitudes toward research were generally positive and were observed to be most favorable among older respondents and those with research experiences. These results may suggest that more widespread use of the RAQ in DS studies could help identify individuals most likely to support enrolling in clinical trials, as well as individuals for whom interventions may be helpful in improving willingness to participate.

## Data Availability

Prior to publication, the study dataset and the survey instrument will be made available in a yet to be determined repository.

## Abbreviations

DS: Down syndrome
AD: Alzheimer disease
RAQ: Research Attitudes Questionnaire
ID: Intellectual disability
